# Population History and Pathways of Spread of the Plant Pathogen *Phytophthora plurivora*

**DOI:** 10.1371/journal.pone.0085368

**Published:** 2014-01-10

**Authors:** Corine N. Schoebel, Jane Stewart, Niklaus J. Gruenwald, Daniel Rigling, Simone Prospero

**Affiliations:** 1 Swiss Federal Institute for Forest, Snow and Landscape Research WSL, Department of Biodiversity and Conservation Biology, Birmensdorf, Switzerland; 2 USDA-ARS-Horticultural Crops Research Laboratory, Corvallis, Oregon, United States of America; 3 Department of Botany & Plant Pathology, Oregon State University, Corvallis, Oregon, United States of America; Agriculture and Agri-Food Canada, Canada

## Abstract

Human activity has been shown to considerably affect the spread of dangerous pests and pathogens worldwide. Therefore, strict regulations of international trade exist for particularly harmful pathogenic organisms. *Phytophthora plurivora,* which is not subject to regulations, is a plant pathogen frequently found on a broad range of host species, both in natural and artificial environments. It is supposed to be native to Europe while resident populations are also present in the US. We characterized a hierarchical sample of isolates from Europe and the US and conducted coalescent-, migration, and population genetic analysis of sequence and microsatellite data, to determine the pathways of spread and the demographic history of this pathogen. We found *P. plurivora* populations to be moderately diverse but not geographically structured. High levels of gene flow were observed within Europe and unidirectional from Europe to the US. Coalescent analyses revealed a signal of a recent expansion of the global *P. plurivora* population. Our study shows that *P. plurivora* has most likely been spread around the world by nursery trade of diseased plant material. In particular, *P. plurivora* was introduced into the US from Europe. International trade has allowed the pathogen to colonize new environments and/or hosts, resulting in population growth.

## Introduction

Global trade has dramatically increased the chances of pathogens to be spread artificially around the world, on or with traded goods [1]–[3]. The introduction of a pathogenic organism into a new area may result in a biological invasion with devastating ecological and economic consequences [3], [4]. After viruses, fungi in a broad sense (including fungal-like organisms, such as oomycetes) are the second most important taxonomic group responsible for plant emerging infectious diseases [5]. Historically, the fungal component of biological invasions has been neglected because of the lack of scientific knowledge on fungal diversity and ecology [6].

Recent invasive diseases with dramatic consequences have gained a lot of attention, e.g. the worldwide amphibian dieback caused by the chytrid fungus *Batrachochytrium dendrobatidis*, where global trading of African clawed frogs (*Xenopus laevis*) for laboratory purposes resulted in dispersal of the pathogen [7]. Other well known invasive fungal diseases include those affecting major food crops, such as the rice blast disease (*Magnaporthe oryzae*, Ascomycota) which cause severe losses of harvests of wheat and barley [8] or the downy mildews of grapevines (*Plasmopara viticola*) and hop (*Pseudoperonospora humili*) [9], [10].

Nonetheless, there are also invasive pathogens with less dramatic consequences that may be difficult to detect. This applies, for example, to invasive pathogens that infect native host species, which are present at low frequencies in specific ecosystems and/or only play a marginal ecological role. Furthermore, pathogens that have established in a non-native ecosystem a long time ago and have, in the meanwhile, become part of this ecosystem are not necessarily considered invasive anymore. Historic records may be helpful, as it is the case for the root fungus *Armillaria mellea* that was introduced to South Africa by Dutch settlers more than 300 years ago, but remained undetected until recently [11]. Finally, the character of invasiveness is difficult to assess in species with a broad natural distribution range and the ability to spread over long distances, as with many fungi do [12].

Several invasive diseases are well known and are being increasingly reported in forest ecosystems worldwide [13]. Current examples include chestnut blight (*Cryphonectria parasitica*) in North America and Europe [14] and, more recently, ash (*Fraxinus* sp.) dieback caused by the ascomycete fungus *Hymenoscyphus pseudoalbidus* in Europe [15]. Many significant recent declines and dieback phenomena in forests have been associated with *Phytophthora* species (Straminipila, Oomycetes), e.g. Sudden Oak Death [16]. Most of these pathogenic species are considered to be exotic in the ecosystems they have invaded and damaged. Specific studies conducted in Europe and North America have clearly shown that the plant nursery trade is a main pathway for the dispersal of *Phytophthora* species [17], [18]. At the genetic level, the most thoroughly investigated *Phytophthora* species in forest ecosystems is probably *P. ramorum* [16]. To date, the global population structure and the pathways of spread of most forest *Phytophthora* species are still poorly understood.

*Phytophthora plurivora,* one of the four species of the former *P. citricola* species complex, is a widespread pathogen in different environments in Europe [19]. In forests, this species acts as a fine root pathogen and is involved in widespread declines of European beech (*Fagus sylvatica*) and oak species (*Quercus* sp.; [20], [21]). In European nurseries, *P. plurivora* is frequently isolated from blighted ornamentals, particularly rhododendrons [22], [23]. *P. plurivora* has also been reported from natural environments (streams, forest soil) in the Eastern and North Central United States [24] as well as in the Western United States [25], [26]. On other continents, *P. plurivora* has only been sporadically found in plantations and nurseries, e.g. in Australia and South Africa. Due to its regular recovery from natural ecosystems in Europe and its rare recovery in other continents, *P. plurivora* is generally assumed to be native to Europe. However, to this date scientific evidence is missing to confirm this hypothesis.

*Phytophthora plurivora* is considered to be a homothallic species [19]. Homothallic species are characterized by the presence of only one mating type and by the production of sexual spores (oospores) by self-fertilization [27]. Accordingly, the formation of oospores is frequently observed in pure cultures of *P. plurivora* [19]. Although homothallism is the most common mode of reproduction in the genus *Phytophthora* [28], the most detailed studies have been conducted on heterothallic species (e.g. *P. ramorum*, *P. infestans*, and *P. cinnamomi*). Therefore, little detailed information is available about the consequences of a homothallic mating system on the expected genetic diversity of *Phytophthora* populations.

With the advent of molecular tools it is now possible to assess whether a pathogen is invasive and determine the most probable center of origin [29]. As species-specific molecular markers are nowadays relatively easy to develop [30], it is highly feasible to use them to reconstruct the main pathways of spread and to investigate the genetic population structure of emerging infectious pathogens [31]–[33]. Population genetic approaches have proven to be valuable for investigating the dispersal of pathogens, their evolutionary history and their epidemiology [34].

The main objective of this study was to quantify the genetic diversity of the global *P. plurivora* population and to use a coalescent approach to determine the demographic history of this species as well as the degree of gene flow among geographic populations. Specifically, we addressed the following questions and related hypotheses: (i) do microsatellite and sequence data support a homothallic mating system in *P. plurivora*? We hypothesized that in that case no traces of recombination should be present in the sequenced genes and that microsatellite data should reveal a high degree of inbreeding; (ii) what are the main directions of gene flow within the global *P. plurivora* population? We hypothesized that the US populations originated from Europe; (iii) is the European *P. plurivora* population geographically structured? Because of the frequent association of *P. plurivora* with nursery trade, we hypothesized that no significant geographic structure is present in Europe; and (iv) what kind of demographic trend (i.e. expansion, reduction, or stability) is detectable in the global *P. plurivora* population? Given the detection of *P. plurivora* in different environments and geographic ranges, we would exclude a population reduction as a demographic trend and hypothesize that the population is either stable or expanding. To answer these questions, we genotyped and sequenced *P. plurivora* isolates derived from Europe and the US.

## Materials and Methods

### Phytophthora plurivora *isolates*

In the present study, a total of 359 *Phytophthora plurivora* isolates from 16 countries (Austria, Belgium, Czech Republic, England, France, Finland, Germany, Hungary, Italy, The Netherlands, Poland, Serbia, Slovenia, Switzerland, Turkey, and the US) were analyzed. The Swiss isolates originated mostly from our culture collection, whereas all other isolates were kindly provided by colleagues. As *P. plurivora* is not a quarantine organism subject to phytosanitary regulations by the Swiss plant protection ordinance (PSV, SR 916.20), a sampling or import permit is not required.

### Phytophthora species identification

Prior to our analyses, the species identity of all isolates was confirmed by sequencing the ribosomal internal transcribed spacer (*ITS*) region and/or the nuclear beta tubulin (*btub*) gene following the procedure described in Blair et al. [35]. PCR amplification, sequencing and sequence assembly were carried out as previously described in Schoebel et al. [36]. Thereafter, sequences of all samples were aligned with the sequences of four reference isolates (CBS 29529 – *P. citricola s.s.*, CBS 18125 – *P. pini*, CBS 124087 – *P. plurivora* and CBS 124094 – *P. multivora*) obtained from the CBS-KNAW fungal biodiversity center’s culture collection (http://www.cbs.knaw.nl/databases/) to confirm species identity.

### Microsatellite genotyping

The 359 isolates were genotyped at 11 *P. plurivora* specific microsatellite loci (PpMs01, PpMs08, PpMs11, PpMs13, PpMs15, PpMs16, PpMs20, PpMs21, PpMs23, PpMs36, and PpMs39) following the published protocol [36]. To increase the quality of the data set, 138 isolates were subsequently excluded from population genetic analyses, either because of incomplete genotyping data available (i.e. no PCR amplicon at one or more loci) or because isolates originated from very small populations (i.e. with less than four multilocus genotypes). Hence, a clone corrected dataset of 221 isolates, each representing a single genotype, was considered for the final analyses.

### Microsatellite analyses

**Figure 1:**
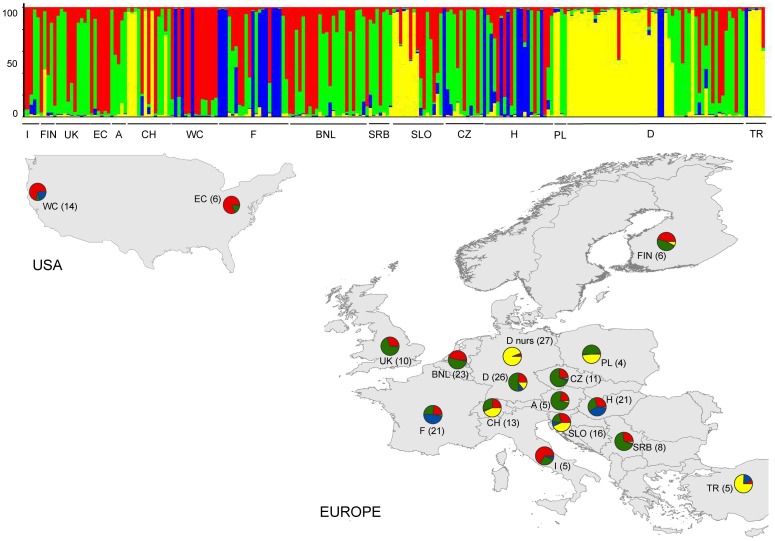
Population clustering of *Phytophthora plurivora*. Genetic clustering (K = 4) of the 16 populations (N = 221 isolates) using 11 microsatellite markers. Each bar represents an individual isolate and colors code the proportion of membership to each cluster. The assignment probability is indicated on the left hand side. Populations are abbreviated as follows (left to right in the bar plot): Italy (I), Finland (FIN), United Kingdom (UK), US East Coast (EC), Austria (A), Switzerland (CH), US West Coast (WC), France (F), Belgium and Netherlands (BNL), Serbia (SRB), Slovenia (SLO), Czech Republic (CZ), Hungary (H), Poland (PL), Germany (D, D nurs), Turkey (TR). D nurs indicates isolates derived from German nurseries only, D all other isolates. Pie charts depict the genetic membership (q-mean) per population, in parentheses the number of isolates  =  multilocus genotypes per population.

For specific analyses (F_ST_, rBarD, and allelic richness, see below), the 221 isolates were combined into 12 geographic populations in order to increase the sample sizes of each respective population, i.e. US-East Coast (EC), US-West Coast (WC), United Kingdom (UK), Finland (FIN), France (F), Belgium and the Netherlands (BNL), Germany (D), Eastern Europe (EEU, with samples from the Czech Republic (CZ), Poland (PL), and Hungary (H)), Alps (with samples from Switzerland (CH) and Austria (A)), Balkans (BAL, with samples from Serbia (SRB) and Slovenia (SLO)), Italy (I), and Turkey (TR; Fig. 1). The 12 geographic populations were also used for gene flow analyses with BAYESASS.

**Population diversity.** For each population, the observed (H_O_) and expected (H_E_) heterozygosities were calculated using the software GENETIX [37]. Population specific allelic richness (Ar) and incidence of private alleles (Pa) were calculated using a generalized rarefaction approach as implemented in the program ADZE [38]. The hypothesis of random mating within populations was tested using the index of association statistics [39]. Specifically, the index rBarD, which corresponds to the index of association I_A_ but is independent from the number of loci considered, was calculated using the R package POPPR [40], [41]. In association tests, rBarD is expected to be zero if populations are freely recombining and significantly greater than zero if there is association between alleles (clonality). The overall genetic differentiation among populations (F_ST_; 1000 randomizations) and pairwise F_ST_-values [42] between populations, for countries as well as regions, were calculated using ARLEQUIN v. 3.1 [43] and GENEPOP [44]. Significant deviations from Hardy-Weinberg equilibrium expectations were evaluated by Fisher’s exact tests, with unbiased P-values (10,000 dememorizations, 100 batches, 5,000 iterations per batch) as implemented in GENEPOP.

**Population history.** The hypothesis of a population expansion was tested using the Microsoft Excel macro KGTESTS [45]. The within-locus k-test [46] was used to compare observed microsatellite allelic distributions with those expected under mutation–drift equilibrium. A negative value in the k-test is indicative of population expansion while positive values indicate population stagnation. The significance of k was determined according to a one-tailed binomial distribution [45]. Moreover, the inter-locus g-test [46] was used to compare observed versus expected allele size variances across all loci. This ratio is expected to be small in a recently expanded population in which allele genealogies show recent coalescence, but large in a population of constant size because of longer histories of variable mutation rates among loci. To determine significance of the test, the g values were compared with the 5% percentile cut off from simulations of g values under constant population size (see Table 1 in [47]). As discussed by Gladieux et al. [31], the power of the g-test to detect recent population expansion is low, especially when the mutation rate across loci is high [48], [49]. Hence, results of the g-test should be interpreted with care.

**Table 1:** Population diversity measures for 12 European and US *Phytophthora plurivora* populations.

Population^a^	N^b^	H_E_ c	H_o_ d	Ar^e^	Pa^f^	rBarD^g^	p-value rBarD^h^
I	5	0.23	0.07	1.44±0.17	0.01±0.01	0.09	0.29
FIN	6	0.17	0.04	1.39±0.23	0.18±0.11	–0.06	0.80
UK	10	0.24	0.12	1.48±0.23	0.11±0.09	–0.05	0.75
EC	6	0.17	0.09	1.33±0.16	0.04±0.03	0.04	0.39
Alps	18	0.33	0.04	1.69±0.23	0.12±0.08	0.04	0.04*
WC	14	0.42	0.10	1.85±0.13	0.40±0.11	0.47	< 0.001***
F	21	0.51	0.18	2.07±0.23	0.32±0.15	0.26	< 0.001***
BNL	23	0.25	0.13	1.47±0.21	0.13±0.07	–0.01	0.68
BAL	24	0.35	0.07	1.75±0.21	0.12±0.06	0.02	0.18
EEU	36	0.46	0.05	2.00±0.21	0.32±0.14	0.16	< 0.001***
D	53	0.33	0.07	1.72±0.22	0.16±0.08	0.10	< 0.001***
TR	5	0.38	0.03	1.77±0.16	0.17±0.09	0.23	0.002**

^a^ Populations are abbreviated as follows: Italy (I), Finland (FIN), United Kingdom (UK), US East Coast (EC), Austria and Switzerland (Alps), US West Coast (WC), France (F), Belgium and Netherlands (BNL), Serbia and Slovenia (Balkans, BAL), Czech Republic, Hungary and Poland (Eastern Europe, EEU), Germany (D) and Turkey (TR).

^b^ Clone corrected data set, thus N equals the number of individuals and the number of multilocus genotypes.

^c^ Expected heterozygosity.

^d^ Observed heterozygosity.

^e^ Allelic richness, mean ± standard error.

^f^ Private alleles, mean ± standard error.

^g^ Index of association.

^h^ Significance intervals for the index of association: *** p ≤ 0.001, ** p < 0.01, * p < 0.05.

In addition to testing for expansion, populations were also tested for evidence of a bottleneck using the software BOTTLENECK v. 1.2.02 [50]. The Wilcoxon-test and the infinite allele model (IAM) of mutations were used. Populations that have recently gone through a severe reduction in the effective population size show a faster reduction in allele number than in heterozygosity. Therefore, the observed heterozygosity is higher than the expected equilibrium heterozygosity as calculated for a population of constant size. For the analyses, data were divided into two populations: Europe (including samples from Turkey) and the US.

**Gene flow.** The software BAYESASS v. 3.0 [51] was used to obtain an estimate of the magnitude and direction of contemporary gene flow between pairs of populations. BAYESASS uses a Monte Carlo Markov Chain (MCMC) algorithm to estimate the posterior probability distribution of the proportion of migrants (M) from one population to another without assuming genetic equilibrium. MCMC chains were run in five independent runs for 100,000,000 generations (10,000,000 burn in) with a sampling frequency of 1,000. To assess the optimal mixing parameters for allele frequencies (a), inbreeding coefficients (f) and migration rates (m), we ran 10 additional short MCMC runs. Short runs consisted of 5,000,000 generations (burn-in 1,000,000) with the same sampling frequency. If the acceptance rate of a run is too high, the chain does not mix well and thus does not explore the state space adequately. By increasing the proposal step size for the mixing parameters a, f and m, the acceptance rate could be decreased. However, mixing parameters could not be fully decreased to the recommended acceptance threshold of 20 – 40%. This might be due to the high degree of inbreeding in our populations (see Results for details). For the final runs, the mixing parameters m = 0.9, a = 0.5 and f = 0.5 were used. For migration rate m = 0.1 was chosen as the relevant cut-off [52]–[54].

**Genotype clustering.** Population assignment tests were carried out using STRUCTURE v. 2.3.3 [55]. STRUCTURE estimates the probability of genotypes being distributed into K number of clusters (K = 1 – n). For all 221 isolates (for details see Table A of File S1), the membership coefficient for every cluster was calculated. An admixture model without prior population information was implemented assuming correlated allele frequencies and using 1,000,000 MCMC sampling repeats (burn-in 100,000). Ten independent runs each for K = 1–10 were carried out. The optimal number of clusters is generally set at the threshold at which the mean logarithm of the probability of the data [Ln P(X|K)] reaches a plateau, i.e. no additional information can be obtained from increasing the number of clusters any further [55]. Nei’s genetic unbiased distances [56] among clusters were calculated as implemented in Tools for Population Genetic Analyses (TFPGA) v. 1.3 [57]. To visualize the genetic relationship among clusters, the resulting distance matrix was used to construct a phenogram based on the unweighted pair-group method of averages (UPGMA) algorithm in TFPGA. Statistical support for phenogram branches was obtained using 1000 bootstrapped samples of the data set.

### Additional sequencing

In addition to *ITS* and *btub*, for a subset of 37 isolates, selected to represent all clusters detected by the STRUCTURE analysis, five other genes were sequenced. These genes were the four nuclear genes *enolase*, heat shock protein 90 (*HSP90*), *TigA* gene fusion protein (*TigA*), the tryptophan biosynthesis gene 1 (*trp1*), and the mitochondrial gene cytochrome c oxidase subunit I (*cox I*). PCR products were amplified using the specific primer sets Fm84 and Fm83 [58] for *cox I*, Enl_for and Enl_rev [35] for *enolase*, HSP90_F1, HSP90_F3, HSP90_R1, and HSP90_R2 [35] for *HSP90*, Tig_for, Tig_F2, Tig_rev, and G3PDH_rev [35] for *TigA*, and Trp1F1, Trp1R1, Trp1F2, and Trp1R2 [18] for *trp1*. PCR reactions were conducted following the respective reference. For detailed methodology see [36].

### Data accessibility

DNA sequences have been submitted to NCBI Genbank (accession numbers KF443812-KF444041) as well as to *Phytophthora* Database (http://www.phytophthoradb.org). For details see Table B of File S1.

### Sequence analyses

JMODEL TEST (available at URL: http://code.google.com/p/jmodeltest2/) and DT-MODSEL [59] were used for the selection of nucleotide substitution models for all genes. DT-MODSEL utilizes PAUP* v. 4.0 [60] to generate a score file and a tree file. Format conversion (.fas to.nex) was done using MESQUITE v. 2.75 (available at URL: http://mesquiteproject.org).

All datasets were checked for evidence of recombination, incompatible sites and violation of neutral evolution using SNAP WORKBENCH [61]. For each gene only haplotypes were used for further analyses. To verify the suitability of each gene for coalescent analyses, its neutrality was estimated using Fu and Li’s D and Tajima’s D values. Potential recombination within each gene was tested using RMIN [62] as implemented in DNASP v. 5.1 [63]. Additional estimates of gene diversity, including nucleotide, sequence, and genetic diversity were calculated with DNASP. Incompatibility matrices [64] were estimated in SNAP CLADE and SNAP MATRIX as implemented in SNAP WORKBENCH. This aimed to visualize incompatible nucleotide sites, such as those arising from recombination or recurrent mutation. Sequences were collapsed into unique haplotypes using SNAP MAP [65] and SITES v. 1.1 [66] by removing indels and incompatible sites.

Genetrees were generated for loci with more than three single nucleotide polymorphisms and without signals of recombination including the *ITS*, *btub*, and *cox I* gene regions with the program GENETREE [67] within the SNAP WORKBENCH package. Additionally, haplotype networks were calculated and visualized for each gene separately using TCS v. 1.2.1 [68].

The software BEAST v. 1.7.2 [69] was used to compute an extended Bayesian skyline plot (EBSP) each for the nuclear genes and for *cox I*, as well as a maximum clade credability tree using sequence information from all seven genes. BEAUTi v. 1.7.2 [69] was used to creat the xml inputfile for BEAST. For this, the clock was set to *cox I* (strict clock), and the following substitution models were chosen: TN93 for *btub*, *HSP90* and *enolase*, HKY for *ITS*, *trp1* and *TigA*. A UPGMA starting tree was chosen as a tree prior and priors were linked for all trees. Furthermore, the option “coalescent: constant size” and a chain length of 250,000,000 (25% burn in) were selected. The prior for the clock rate was set to lognormal.

## Results

### Population diversity and structure

In the entire *P. plurivora* population, a total of 109 alleles were detected across the 11 microsatellite loci. All loci were polymorphic with two (locus PpMs11) to 30 (locus PpMs01) observed alleles. Allelic richness (Ar) and private alleles (Pa) were highest for populations US West Coast (Ar: 1.8; Pa: 0.4), France (Ar: 2.1; Pa: 0.32), and Eastern Europe (Ar: 2.0; Pa: 0.32). The lowest Ar and Pa values were observed in the US East Coast population (Ar: 1.3; Pa: 0.04; Table 1). Locus-specific observed heterozygosity varied between 0 (locus PpMs13) and 0.5 (locus PpMs23) for all populations as well as in the entire population at all loci. However, for locus PpMs10 the observed heterozygosity was significantly (p < 0.05) lower than the expected heterozygosity (Table 1). Observed heterozygosity (H_o_) values ranged between 0.03 (Turkey) and 0.18 (France), with a mean value of 0.08 across all 12 populations. The index of association rBarD was significantly different than zero in six geographic populations (Alps, East Coast, France, Eastern Europe, Germany and Turkey), which indicates a significant deviation from random mating. In the other six populations, based on the index of association statistics the hypothesis of random mating could not be rejected.

F_ST_-statistic analysis revealed an overall population differentiation of F_ST_ = 0.102. F_ST_-values between pairs of populations varied between 0.01 (BNL and UK) to 0.38 (BNL and TR), but none of the F_ST_ values were significant (Table C, File S1).

Significant contemporary gene flow from Belgium and the Netherlands (BNL) to both the US East (EC) and West Coast (WC) was detected for the microsatellite data using BAYESASS. Furthermore, contemporary gene flow could be detected from BNL to the UK and to France (F). Additionally, significant migration from Germany to the Alps (Switzerland), to Eastern Europe (Czech Republic), to the Balkans (Slovenia) and to Turkey could be detected (Table 2).

**Table 2:** Bayesian assessment of migration within and among sampling localities of *Phytophthora plurivora* implemented in BAYESASS [51].

Sink/	Source population											
	I	FIN	UK	EC	Alps	WC	F	BNL	BAL	EEU	D	TR
I	**0.69**							0.05			0.09	
	(0.65 – 0.73)							(–0.02 – 0.11)			(0.01 – 0.17)	
FIN		**0.69**						0.07			0.08	
		(0.65 – 0.72)						(0.00 – 0.14)			(0.01 – 0.15)	
UK			**0.68**					*0.12*			0.06	
			(0.65 – 0.71)					(0.03 – 0.21)			(–0.02 – 0.15)	
EC				**0.69**				*0.10*			0.05	
				(0.65 – 0.72)				(0.02 – 0.17)			(–0.01 – 0.12)	
Alps					**0.68**	0.01					*0.19*	
					(0.66 – 0.70)	(–0.01 – 0.03)					(0.13– 0.24)	
WC						**0.68**	0.06	*0.14*				
						(0.65 – 0.71)	(0.01 – 0.11)	(0.08 – 0.20)				
F							**0.77**	*0.11*				
							(0.72 – 0.82)	(0.05 – 0.16)				
BNL								**0.89**				
								(0.84 – 0.95)				
BAL									**0.7**		*0.20*	
									(0.66 – 0.69)		(0.15 – 0.25)	
EEU							0.06	0.05		**0.67**	*0.16*	
							(0.03 – 0.10)	(0.01– 0.10)		(0.66 – 0.69)	(0.10 – 0.21)	
D											**0.93**	
											(0.89 – 0.96)	
TR											*0.12*	**0.69**
											(0.04 – 0.19)	(0.65 – 0.73)

For each population, numbers are given as the level of gene flow (proportion of migrants per generation) from the source population on the right (column headings) into the sink population on the left (row headings). Bold numbers along the diagonal are the proportion of non-migrants (self-recruitment). 95% confidence intervals are given in parentheses. Migration rates greater than 0.1 are underlined and italicized. Empty cells represent mean proportions of lower than 0.050. Populations are abbreviated as follows (left to right): Italy (I), Finland (FIN), United Kingdom (UK), US East Coast (EC), Switzerland and Austria (Alps), US West Coast (WC), France (F), Belgium and Netherlands (BNL), Serbia and Slovenia (Balkans, BAL), Czech Republic, Hungary and Poland (Eastern Europe, EEU), Germany (D) and Turkey (TR).

**Figure 2:**
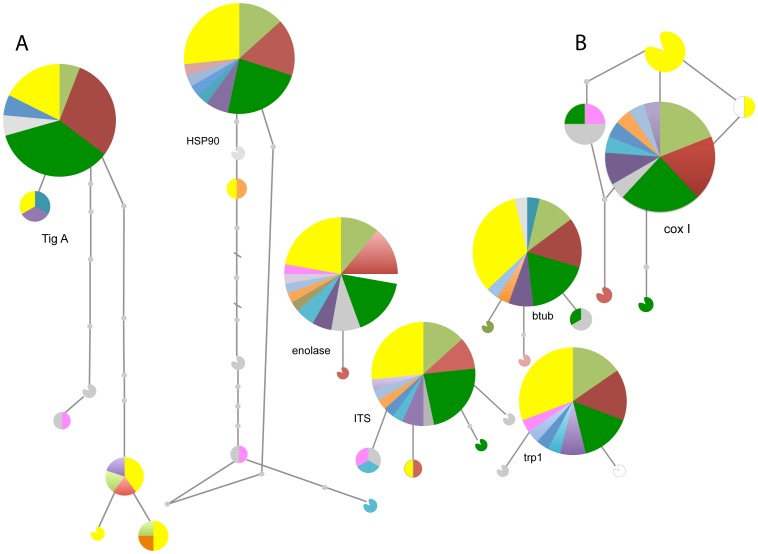
Haplotype networks for *Phytophthora plurivora*. (a) Haplotype networks for the 6 nuclear genes: transcriptomal gene fusion protein (*TigA*, 33 isolates), heat shock protein 90 (*HSP90*, 36 isolates), *enolase* (36 isolates), tryptophan biosynthesis gene 1 (*trp*, 28 isolates), internal transcribed spacer (*ITS*, 37 isolates) and the beta tubulin gene (*btub*, 32 isolates). (b) Haplotype network of the mitochondrial gene cytochrome C oxidase subunit I (*cox I*, 33 isolates). Each circle represents a different haplotype, small grey circles represent unsampled, intermediate, haplotypes and colors represent the affiliation to a certain population. Color code: light green – Belgium, white – Czech Republic, purple – Finland, grey – France, green – Germany, turquois – Hungary, dark blue – Italy, orange – Netherlands, mauve – Slovenia, light blue – Serbia, red – Switzerland, pink – Turkey, and yellow – USA.

As shown in Fig. 2, the 37 isolates sequenced belonged to a maximum of 7 different haplotypes (details Table D, File S1). The highest number of haplotypes was observed in the nuclear gene *TigA* (7 haplotypes), followed by the nuclear gene regions btub and *HSP90* (6 haplotypes), and by the mitochondrial gene *cox I* (6 haplotypes, but 5 haplotypes in the coalescent analyses due to one incompatible site at position 824). The least variation was observed for the nuclear gene regions enolase (2 haplotypes) and *trp1* (3 haplotypes). In all gene regions, a dominant haplotype was detected that was accompanied by a few, less common haplotypes. In all genes, no clustering of haplotypes according to their geographic origin was observed (Fig. 2, Fig. S1). According to our coalescent analyses the ancestral haplotype for the *ITS* region could be found in France, Hungary and Turkey, and for *cox I* in Germany. For *btub*, all haplotypes diverged at the same time (Fig. S1). In all nuclear gene regions, the North American isolates belonged, together with most European isolates, to the most common haplotype. On the contrary, for the *cox I* gene the most common haplotype was only found across the European samples and the North American isolates belonged to different haplotypes. For *TigA, HSP90* and *ITS*, US isolates are both present within the central haplotype as well as in other haplotype(s). On the other hand, for *btub* and *trp1* US isolates are only characterized by the most common, central haplotype (Fig. 2).

None of the gene regions showed evidence of selection, with p-values of Tajima’s D unable to reject the null hypothesis of neutral evolution (Table 3). Sequence diversity between gene regions was comparable among the *ITS, cox I*, and *btub* gene regions, ranging from θ = 0.99 to 1.46. The *enolase* and *trp1* genes showed a lower diversity with θ = 0.24 and 0.51, respectively. Sequence diversity was highest for the *HSP90* and *TigA* regions (θ = 1.91 and 2.69, respectively), but evidence for recombination was also detected in these two loci (Table 3). Recombination was not detected within the other five loci.

**Table 3:** Genetic diversity observed per locus in *Phytophthora plurivora* isolates from Europe and the US.

gene	Taj D	p Taj D	no. seqs	no. HT	no. sites	no. poly	π	θ seq	k	Rm
*cox I*	–1.81	>0.1	33	6	1083	6	0.0009	1.46	1.01	0
*btub*	–1.60	>0.1	32	4	1058	4	0.0003	0.99	0.36	0
*enolase*	–1.13	>0.1	36	2	1125	1	0.0001	0.24	0.05	0
*HSP90*	–0.97	>0.1	36	6	1528	8	0.0008	1.92	1.25	1
*ITS*	–1.68	>0.1	37	5	690	5	0.0006	1.19	0.42	0
*TigA*	0.29	>0.1	33	7	1497	11	0.0020	2.69	2.94	2
*trp1*	–1.51	>0.1	28	3	492	2	0.0003	0.51	0.14	0

Abbreviations are as follows: Taj D (Tajima’s D), p Taj D (p value Tajima’s D), no. seqs (number of sequences), no. HT (number of haplotypes), no. sites (number of sites), no. poly (number of polymorphic sites), π (pi; nucleotide diversity), θ seq (theta, per sequence), k (average number of nucleotide differences), Rm (minimum number of recombination events).

### Clustering and population assignment tests

STRUCTURE analysis showed that Ln P(X\K) increased more or less constantly until K = 10, without reaching a clear plateau (Fig. S2). Therefore, in order to detect the optimal number of clusters to best represent our data, we considered the change of ΔK (i.e. the ad hoc quantity related to the second order rate of change of the log probability of data) with respect to the number of clusters [70]. The value of ΔK decreased until K = 4 and then remained constant (Fig. S2). Thus, we decided that the presence of four genetic clusters could best explain the worldwide population structure of *P. plurivora.* These clusters did not correspond to geographic groups of isolates (Fig. 1). The first cluster (yellow color in Fig. 1) mainly comprised isolates from German and Swiss nurseries, as well as isolates from Slovenian and Turkish forests. Two isolates from Poland and one from Finland also added to this cluster. To the second cluster (blue) belonged only forest isolates, the great majority of them originating from alder stands. This cluster was common in France and Hungary and was also found in Turkey and Slovenia (one isolate each), Germany (two isolates), and the US-West Coast (three isolates). The third (red) and fourth (green) clusters comprised both forest and nursery isolates from most geographic populations.

**Figure 3:**
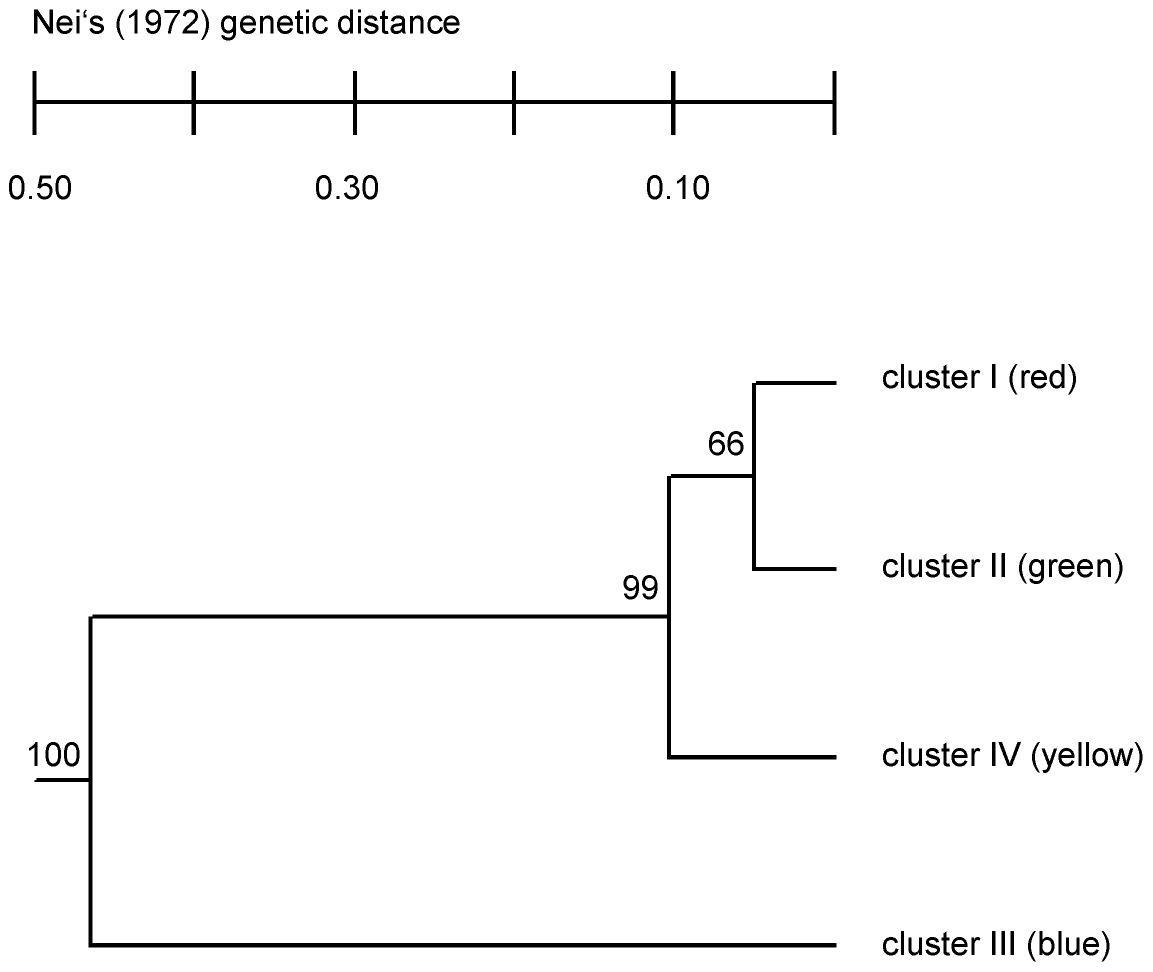
Phenogram of the four *Phytophthora plurivora* clusters detected with STRUCTURE. The phenogram was constructed using the unweighted pair-group method of averages (UPGMA) algorithm based on Nei’s unbiased genetic distance [56] of clusters. Statistical support for branches was obtained using 1000 bootstrapped samples of the data set. Clusters match the color code in Fig. 1.

The UPGMA Phenogram based on the Nei’s unbiased genetic distance [56] indicates that the green and red groups (cluster 1 & 2) are more closely related to each other than to the yellow (cluster 3) and blue (cluster 4) groups. This is well supported by the bootstrap values given next to each node (Fig. 3).

### Demographic history

BOTTLENECK analysis using the Wilcoxon test revealed no significant bottleneck signals either in the US (p = 0.38) or in the European (p = 0.91) population for the microsatellite data. The mode shift test showed a normal L-shaped distribution for the European population and a shifted mode, which is indicative of a bottleneck, for the US population. A signature of recent population expansion was detected in the European as well as in the US *P. plurivora* population by conducting within-locus k tests. A significant number of negative k values were observed for 10 of the 11 loci (k test p = 0.004; g = 3.4) for the pooled European samples and for all 20 US samples pooled (k test p = 0.004; g = 3.78). The inter locus g statistic was higher than 1 in both populations (Europe: g = 3.4; US: g = 3.78), which is indicative of no population expansion.

**Figure 4:**
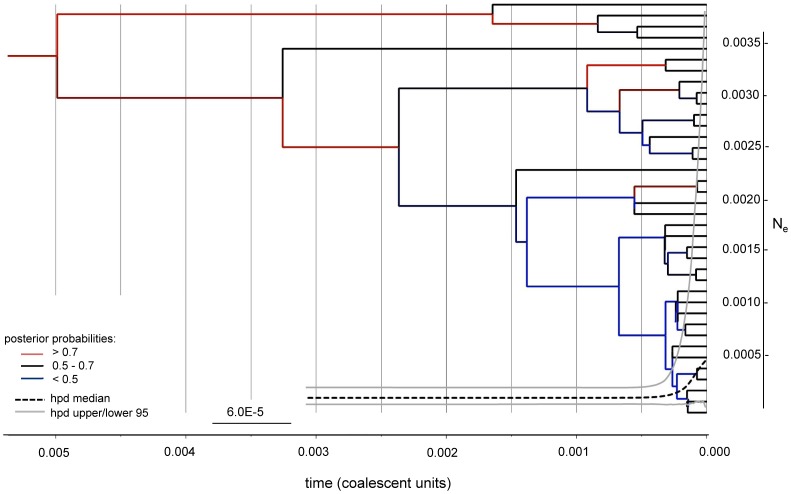
Maximum clade credibility tree for European and US *Phytophthora plurivora* isolates. The tree was depicted for six nuclear genes (*ITS, btub, enolase, TigA, trp1* and *HSP90*) and the mitochondrial gene *cox I.* Extended Bayesian skyline plot (EBSP) for the six nuclear genes (*ITS, btub, enolase, TigA, trp1* and *HSP90*). Posterior probabilities are indicated in red (>0.7), black (0.5 – 0.7) and blue (<0.5). Time is shown in coalescent units on the x-axis. The highest posterior density (hpd) median is depicted as a black dashed line and the hpd upper/ lower 95 limits as grey solid line. The scale bar on y-axis indicates the effective population size (N_e_).

Conducting Bayesian phylogenetic multi-gene analyses (maximum credibility tree as well as EBSP, Fig. 4) on non-neutral nuclear and mitochondrial sequence data, we detected a recent increase in effective population size and no bottlenecks. BEAST analysis showed good convergence with 130 million of generations. Moreover, all of the ESS parameter had values > 400.

## Discussion

Like most *Phytophthora* species, *P. plurivora* has a homothallic (i.e. self-fertile) mating system [19]. Previous studies have indicated that selfing reduces genetic diversity and effective population size and hence considerably increases the level of homozygosity relative to sexual populations [71]–[73]. Therefore, we hypothesized that in *P. plurivora* low levels of recombination should be present in the sequenced genes and that microsatellite data should reveal a high degree of inbreeding. Our results provide evidence of low levels of recombination given that five out of seven genes show no recombination (exception: *TigA* with two recombination events and *HSP90* with one event) and a significant excess of homozygosity at all microsatellite loci. These results are further supported by our rejection of the null hypothesis of no linkage among loci in most populations as measured by the index of association.

High degrees of homozygosity were previously observed in *P. alni* subsp. *uniformis* [74] and in *P. sojae* [75], which are, to our knowledge, the only other homothallic *Phytophthora* species that have been investigated so far using population genetic tools. However, *P. alni* subsp. *uniformis* and *P. sojae* present important ecological differences compared to *P. plurivora*. The first is most likely exotic to Europe and, thus, the low genetic diversity and the homozygosity excess observed at some loci may be a consequence of the fixation or loss of specific alleles after its introduction into the new continent. The latter is a pathogen of soybean in agricultural fields whose origin remains uncertain [75], [76]. *P. plurivora* is expected to be native to Europe and the significant deficit of heterozygosity detected at all loci could support this hypothesis. In fact, if we consider that in self-fertile, diploid species heterozygosity should decrease by 50% in each generation [72], there should be hardly any heterozygous loci detectable in native populations of homothallic *Phytophthora* species.

Different modes of homothallism are described in literature, including two nuclei in one spore, two either linked or unlinked mating types in one nucleus, two mating types in one nucleus that undergo mating type switching, or only one mating type present at all [27], [77]. In general, Phytophthoras are considered homothallic if they are able to form oospores (i.e. sexual spores) in single cultures. However, at present no detailed information on the mode of homothallism in the genus *Phytophthora* exists. The presence of two genes (*TigA* and *HSP90*) showing clear traces of recombination events might suggest that in *P. plurivora* outcrossing, although rare, is or was possible. The potential to outcross was previously described for the homothallic species *Pythium ultimum* (oomycete) and for *P. sojae* [71], [78].

In plant taxa, self-fertilization is commonly expected to be an evolutionary dead end as it is accompanied by the loss of genetic diversity and, thus, by a reduction of the chances to adapt to new and/or changing environments. For this reason, selfing lineages often go extinct, whereas new lineages are started by outcrossing precursors [79], [80]. Although the self-fertilization as a dead end (SEDE) hypothesis was formulated more than 50 years ago [80], we still do not know whether this hypothesis also applies to fungi and oomycetes. Most *Phytophthora* species are plant parasites and, thus, for survival rely on a host. For host species, vegetative reproduction and self fertilization is considered disadvantageous as clonal hosts, lacking genetic variability created through recombination, face an evolutionary disadvantage. On the contrary, outcrossing hosts may escape the pressure imposed by coevolving parasites through sexual recombination (Red Queen Hypothesis; [81], [82]). In parasites, a high genetic diversity allows a rapid adaptation to changing host defences (antagonistic co-evolution), which would suggest that selfing may have evolutionary disadvantages for both the host and the parasite. Nonetheless, a recent review, [79] discussed the fact that the evolution of self-fertilization might purge harmful mutations which should reduce inbreeding depression. Furthermore, selfing allows a successful reproduction without the presence of an appropriate mating partner. This option may be particularly useful once an organism is founding a population in a novel geographical area.

Our sequence data indicate that the origin of *P. plurivora* is mostly likely Europe rather than the US, as sequence diversity in European populations is higher than in US populations. In fact, theory predicts that ancestral populations should show increased polymorphism compared to more recent populations [83]. Among 33 *P. plurivora* individuals, we identified six mitochondrial haplotypes, four of them only present in Europe, one only found in the US, and one shared between the US and the Czech Republic. The most common haplotype (central haplotype in Fig. 2, see also Table D, File S1), which only occurs in Europe, does not seem to be the ancestral haplotype for *ITS* and *cox I* based on gene genealogy (Fig. S1). Its broad occurrence may indicate that this haplotype was particularly frequent in plant nurseries and has been spread around via international trade of plant material. The two most likely ancestral haplotypes were, however, found amongst European isolates only.

Microsatellite data also indirectly support a European origin of *P. plurivora*. Migration analyses conducted with the software BAYESASS detected unidirectional gene flow from Europe to the US and not *vice versa*. The presence of a bottleneck in the US *P. plurivora* population was tested for sequence as well as for microsatellite data. For sequence data (both nuclear and mitochondrial sequences) we did not detect any sign of recent bottlenecks. Conducting two specific tests for microsatellite data using the software BOTTLENECK, however, we obtained contradictory results; on one hand, the Wilcoxon test indicated no visible traces of bottleneck, whereas on the other hand, the mode shift test suggested a significant bottleneck. The structure of the data set and the assumptions for the two tests may explain this particular situation. A bottleneck might not be detected if sample sizes are small, the individuals used are not entirely representing the populations, or if the population subject to a bottleneck is not completely isolated. Additionally, one assumption for these tests is that loci are under Hardy Weinberg equilibrium, which was not the case in the present study. Hence, larger samples sizes, especially for the US populations, would be needed to increase the chance of detecting bottleneck effects with microsatellite markers. In order to draw definite conclusions about the centre of origin of *P. plurivora*, further samples from other continents should be analysed, as the present study only includes samples from Europe and North America. Furthermore, the possiblity of an initial introduction to Europe from elsewhere and then from Europe to the US should also be considered.

Based on our analyses, *P. plurivora* has been introduced into the US from Europe, most probably from Belgium and the Netherlands. No significant gene flow was detected between the two US populations, which suggests that two independent introductions from Europe most likely occurred. In mainland Europe, Germany seems to have been the most important *P. plurivora* source for the Alps, the Balkans, and Eastern Europe. Populations in Finland and Italy show low levels of gene flow with other European *P. plurivora* populations, but this might be associated with the small samples size of these specific populations. Due to significant differences in sample sizes between countries, we cannot exclude a bias in the migration analysis towards countries with large sample sizes. Therefore, to definitely confirm our hypotheses about gene flow among and from European countries to North America, a larger number of samples from both forests and nurseries from each country should be analysed. Our findings concur with previous studies that found that nursery trade is an important source for the spread of plant diseases. According to Liebhold et al. [3], about 70% of the non-native forest pests established in US forests have been introduced through the trade of live plants. Goss et al. [18], [84] showed that *P. ramorum* has been dispersed over long distances by means of nursery trade and shipments of infected host plants across the US and the US-Canadian border, as well as from Europe to the US. The Netherlands, Belgium and Germany are important producers and exporters of ornamentals worldwide [85]. Amongst the ornamental plants cultivated and traded, rhododendrons play a major role and are a main host of *P. plurivora* [23], [86]. Our hypothesis that *P. plurivora* spread within Europe and from Europe to the US through infected plants coming from these three countries may therefore be well founded. Several US isolates considered in this study originated from natural environments (i.e. streams, ponds, forest soil) and residential areas, indicating that *P. plurivora* has already become established in the wild, both on the East and West Coast of the US. In a study on the fungal wheat pathogen *Mycosphaerella graminicola* [87], it was suggested that microsatellite loci with recently emerged rare alleles are particularly useful to detect migration events. Accordingly, we may speculate that in our study the gene flow estimates based on microsatellite data could reflect the most recent gene flow events. Assuming that *P. plurivora* has spread mainly through nursery trade, we could hypothesize that the gene flow detected occurred during the past 50 to 100 years. This time frame coincides with global commercial trade expansion and when nursery trade between European countries as well as between Europe and the US has flourished.

According to the high levels of gene flow observed, no clear pattern of geographic structure could be detected within the *P. plurivora* population in Europe. The European isolates analyzed belonged to four divergent microsatellite clusters. Only three and two of them were present on the West and East Coast of the US, respectively. The lineage completely missing in the US was associated with *P. plurivora* isolates obtained from German and Swiss nurseries, as well as with a few isolates originating from Turkish and Slovenian forests. Noteworthy, all isolates recovered from alder (*Alnus* sp.) stands belonged to the same lineage and were detected both in Europe and the US West Coast. By performing inoculation test, it would be possible to verify whether *P. plurivora* genotypes from this specific lineage are better adapted to alder species than genotypes from the other lineages.

In conclusion, our genetic analyses support a homothallic mating system in the plant pathogen *P. plurivora.* Moreover, they show that this species was most likely introduced from Europe (Belgium and the Netherlands) to both the East and West coasts of the US. In mainland Europe, *P. plurivora* may have primarily been spread from German nurseries. The importance of human-mediated international trade for the dispersal of this pathogen is also reflected by the lack of geographic structuring in the *P. plurivora* population sampled in this study. None of the four genetic clusters detected among the analyzed samples corresponded to a specific geographic region. A recent population expansion was detected, which might be due to the human mediated establishment of *P. plurivora* in new environments or on new host plants. While strict regulations exist for quarantine organisms in international plant trade (e.g. inspections, monitoring), indigenous pathogens and pests are usually not subject to such policies. Our analyses, however, indicate that the international plant trade is a major pathway of spread also for non-invasive plant pathogens. Therefore, we would like to emphasize that the unintentional spread of dangerous but non-regulated organisms with diseased plant material should not be underestimated. Similarly to quarantine organisms, non-regulated pathogens may negatively affect local ecosystems and long-term consequences for biodiversity still remain unknown.

## Supporting Information

File S1 **Includes supplementary tables A, B, C and D.** Table A: Origin and supporting information on the *Phytophthora plurivora* isolates used in the STRUCTURE analysis. Table B: Origin, gene bank accession numbers and supporting information on the *Phytophthora plurivora* isolates used in the coalescent analyses. Table C: FST values between all population pairs of European and US *Phytophthora plurivora* isolates. Table D: Supplementary information on the *Phytophthora plurivora* isolates used in the Haplotype Network analysis.
(DOCX)

Figure S1 **Gene genealogy for** ***Phytophthora plurivora.*** Coalescent-based gene genealogy for the three genes (a) *ITS* (37 sequences), (b) *cox I* (33 sequences) and (c) *btub* (32 sequences) generated using GENETREE [67], which assumes no recombination. Each genealogy is scaled to time to the most recent common ancestor (TMRCA) of 1.0 for each locus. Timescale is in coalescent units of effective population size. Mutations are labelled by their location in the sequence. Letters represent haplotypes and numbers underneath indicate the number of isolates that share that specific haplotype. The ancestral haplotype was found in (a) France, Hungary and Turkey and (b) Germany, while for *bub* (c) all haplotypes diverged at the same time. For (c) the oldest haplotype was selected by GENETREE based on likelihood scores. Note: At position 824 of the *cox I* region there was a incompatible site, leading to 5 haplotypes above, while for the analyses in Fig. 2 and Table 3 we indicate 6 haplotypes.
(TIF)

Figure S2 **Additional information on STRUCTURE analysis.** Membership to STRUCTURE [55] groups (K = 2 - 9) of *Phytophthora plurivora* isolates sampled from 16 study populations. (a) Population assignment barplots, (b) Change (mean over 10 runs ± standard deviation) of L(K) (i.e. posterior probability of the model according to the number of genetic clusters tested); (c) change in Δ K (i.e. ad hoc quantity related to the order rate of change of L(K) with respect to the number of genetic clusters tested). The chosen number of clusters was K = 4 (red frame).
(EPS)
