# Correction: Population History and Pathways of Spread of the Plant Pathogen *Phytophthora plurivora*

**DOI:** 10.1371/journal.pone.0105259

**Published:** 2014-08-06

**Authors:** The PLOS ONE Staff

The third author’s name is spelled incorrectly. The correct name is: Niklaus J. Grünwald. The correct citation is: Schoebel CN, Stewart J, Grünwald NJ, Rigling D, Prospero S (2014) Population History and Pathways of Spread of the Plant Pathogen *Phytophthora plurivora*. PLoS ONE 9(1): e85368. doi:10.1371/journal.pone.0085368
