# Supplementary material for: Population History and Pathways of Spread of the Plant Pathogen Phytophthora plurivora
Source: PLoS One. 2014 Jan 10;9(1):e85368. doi: 10.1371/journal.pone.0085368 (PMC3888410; doi:10.1371/journal.pone.0085368)
Supplement: File S1 — Includes supplementary tables A, B, C and D. Table A: Origin and supporting information on the Phytophthora plurivora isolates used in the STRUCTURE analysis. Table B: Origin, gene bank accession numbers and supporting information on the Phytophthora plurivora isolates used in the coalescent analyses. Table C: FST values between all population pairs of European and US Phytophthora plurivora isolates. Table D: Supplementary information on the Phytophthora plurivora isolates used in the Haplotype Network analysis. (DOCX) [file pone.0085368.s001.docx]

**Supplementary information file S1**

**Table A:** Origin and supporting information on the 221 *Phytophthora plurivora* isolates used in the STRUCTURE analysis (clone corrected data set).

| **No in STRUCTURE analysis** | **Our ID** | **Previous ID** | **In coalescent analysis** | **Country** | **Region** | **Host plant** | **Sample type** |
| --- | --- | --- | --- | --- | --- | --- | --- |
| 1 | CS428 | FAGG4x3 |  | Italy _Arola | IT | *Fagus silvatica* | Forest, soil |
| 2 | CS426 | CS1T17G1(3) |  | Italy _Carbagnano | IT | Rural road | Forest, soil |
| 3 | CS427 | 6G3T9.5(7°) | coalescent | Italy _Carbagnano | IT | *Castanea sativa* | Forest, soil |
| 4 | CS425 | 81LT5G2.1(4) |  | Italy _Rieti | IT | *Castanea sativa* | Forest, soil |
| 5 | CS142 | 5481 |  | Finland | SCAN | *Rhododendron sp.* | unknown |
| 6 | CS143 | 450 | coalescent | Finland | SCAN | *Syringa* | unknown |
| 7 | CS144 | 444 | coalescent | Finland | SCAN | unknown | unknown |
| 8 | CS146 | 441 |  | Finland | SCAN | *Rhododendron sp.* | unknown |
| 9 | CS147 | 411 |  | Finland | SCAN | *Rhododendron sp.* | unknown |
| 10 | CS281 | 1594/3 |  | GB_Scotland | UK | *Rhododendron sp.* | unknown |
| 11 | CS282 | 1594/4 |  | GB_Scotland | UK | *Rhododendron sp.* | unknown |
| 12 | CS283 | 511 |  | GB_Scotland | UK | *Rhododendron sp.* | unknown |
| 13 | CS284 | 1445 |  | GB_Scotland | UK | *Pieris spp.* | unknown |
| 14 | CS285 | 1404 |  | GB_Scotland | UK | *Rhododendron sp.* | unknown |
| 15 | CS286 | 817 |  | GB_Scotland | UK | *Rhododendron sp.* | unknown |
| 16 | CS287 | 2427/1 |  | GB_Scotland | UK | *Syringa vulgaris* | unknown |
| 17 | CS288 | 00584/5a |  | GB_Scotland | UK | *Rhododendron sp.* | unknown |
| 18 | CS376 | P33/04 |  | GB_Suffolk | UK | *Sambucus tenuifolium* | Nursery |
| 19 | CS569 | SCRP136, CIT7 |  | UK | UK | *Taxus sp. + soil* | Yew Soil |
| 20 | CS566 | SCRP132, CIT3 |  | UK_England | UK | *Rubus idaeus* | Raspberry |
| 21 | CS608 | W.004 | coalescent | USA_WI | US_E | *Acer saccharum* | not known, residence site |
| 22 | CS610 | W.006 |  | USA_WI | US_E | *Acer platanoides* | not known, residence site |
| 23 | CS612 | W.119 |  | USA_WI | US_E | *Acer platanoides* | plant-crown, residence |
| 24 | CS581 | NY.117 |  | USA_NY | US_E | *Rhododendron x catawbiense* | plant root |
| 25 | CS582 | NY.118 |  | USA_NY | US_E | *Rhododendron x catawbiense* | plant root |
| 26 | CS611 | W.019 | coalescent | USA_WI | US_E | *Rhododendron x catawbiense* | not known, nursery |
| 27 | CS341 | none |  | Austria_Vbg | ALPS | *Fagus sylvatica* | unknown |
| 28 | CS343 | none |  | Austria_Vbg | ALPS | *Quercus sp.* soil/ *Quercu*s bait | close to street |
| 29 | CS344 | none |  | Austria_Vbg | ALPS | *Quercus sp.* soil/ *Rhododendron*bait | close to street |
| 30 | CS345 | none |  | Austria_Vbg | ALPS | *Quercus sp.* soil/ *Quercu*s bait | close to street |
| 31 | CS089 | PI 24 |  | Switzerland | ALPS | *Rhododendron sp.* | nursery |
| 32 | CS092 | PI 29 |  | Switzerland | ALPS | *Rhododendron Hirsutum* | nursery |
| 33 | CS096 | PI 33 | coalescent | Switzerland | ALPS | *Rhododendron Roseum elegans* | nursery |
| 34 | CS110 | PI 65 |  | Switzerland | ALPS | *Rhododendron Inkarho* | nursery |
| 35 | CS191 | MK 303 |  | Switzerland | ALPS | unknown | unknown |
| 36 | CS192 | none | coalescent | Switzerland | ALPS | unknown | unknown |
| 37 | CS073 | 273 | coalescent | Switzerland_ Geneve | ALPS | Acer platanoides | unknown |
| 38 | CS114 | 174 | coalescent | Switzerland_Brissago | ALPS | *Rhododendron sp.,* soil | unknown |
| 39 | CS075 | 294 |  | Switzerland_Etoy | ALPS | Soil via apple | unknown |
| 40 | CS622 | 189 | coalescent | Switzerland_Oppens | ALPS | apple | unknown |
| 41 | CS624 | 197 |  | Switzerland_Oppens | ALPS | unknown | soil |
| 42 | CS538 | CIT 100 |  | Switzerland_Rueschlikon | ALPS | *Q. robur* | unknown |
| 43 | CS074 | 305 |  | Switzerland_Ticino | ALPS | Rhododendron sp., roots | unknown |
| 44 | CS087 | 302 |  | Switzerland_Zufikon | ALPS | soil sample | unknown |
| 45 | CS173 | 120-W-1.17 | coalescent | USA_OR | US_W | Stream | unknown |
| 46 | CS174 | 127-W-2.11 |  | USA_OR | US_W | Stream | unknown |
| 47 | CS176 | 221-W-2.3 |  | USA_OR | US_W | Stream | unknown |
| 48 | CS177 | WA5.1-101403 |  | USA_OR | US_W | Stream | unknown |
| 49 | CS180 | 05-02 | coalescent | USA_OR | US_W | Nursery Foliage | unknown |
| 50 | CS181 | 05-1095-8B |  | USA_OR | US_W | Soil | unknown |
| 51 | CS185 | PC02-747 | coalescent | USA_OR | US_W | unknown | unknown |
| 52 | CS258 | PCT-07-015 | coalescent | USA_OR | US_W | *Rhododendron 'pink'* | leaf |
| 53 | CS271 | JP-08-166 |  | USA_OR | US_W | substrate | soil/substrate |
| 54 | CS256 | JP-07-0310 | coalescent | USA_OR | US_W | *Rhododendron sp.* | pottingmedium |
| 55 | CS260 | JP-08-113 | coalescent | USA_OR | US_W | *Pieris japonica* | roots |
| 56 | CS263 | JP-08-164 |  | USA_OR | US_W | *Rhododendron sp.* | potting medium |
| 57 | CS261 | JP-08-124 |  | USA_OR | US_W | pond | water |
| 58 | CS264 | PCT-09-003 | coalescent | USA_OR | US_W | unknown | soil |
| 59 | CS188 | LNPV125 |  | France | F | unknown | unknown |
| 60 | CS190 | LNPV127 |  | France | F | unknown | unknown |
| 61 | CS290 | 8D5-1 |  | France | F | probably *Quercus robur* | forest |
| 62 | CS291 | AA6 |  | France_Sélestat | F | *Quercus robur* | forest |
| 63 | CS292 | AC4 |  | France_Sélestat | F | *Quercus robur* | forest |
| 64 | CS293 | AD1 |  | France_Sélestat | F | *Quercus robur* | forest |
| 65 | CS294 | AK2 |  | France_Mulhouse | F | *Quercus robur* | forest |
| 66 | CS295 | DU.L8 |  | France_Champenoux | F | *Quercus robur* | forest |
| 67 | CS296 | EQ.L18 |  | France_Champenoux | F | *Quercus robur* | forest |
| 68 | CS297 | FM.1 |  | France_Filain | F | *Quercus robur* | forest |
| 69 | CS298 | I.POC.5 |  | France_Sélestat | F | *Quercus robur* | forest |
| 70 | CS299 | Rhodo.2 |  | France | F | *Rhododendron* | unknown |
| 71 | CS300 | NF11 |  | France_Mersuay | F | *Quercus robur* | forest |
| 72 | CS301 | CIT4 |  | France_Gizay | F | *Quercus sp.* soil | forest |
| 73 | CS303 | CIT3 | coalescent | France_Bazoilles_sur_Meuse | F | *Alnus glutinosa* | riparian forest |
| 74 | CS304 | H1 |  | France_Sélestat | F | *Quercus robur* | forest |
| 75 | CS305 | PHY15.1 |  | France_Oermingen | F | *Alnus glutinosa* | riparian forest |
| 76 | CS306 | PHY8.5 | coalescent | France_Baerendorf | F | *Alnus glutinosa* | riparian forest |
| 77 | CS307 | 08DUR18 |  | France_Saint-Didier | F | *Alnus incana* | riparian forest |
| 78 | CS308 | CIT1 | coalescent | France_Plouigneau | F | *Rhododendron cv Scintillation* | Nursery |
| 79 | CS533 | CIT 65 |  | France_Illwald | F | *Q. robur* | soil |
| 80 | CS231 | PD 20017841 |  | Belgium | BNL | *Laurus* | unknown |
| 81 | CS309 | D/10/2122 |  | Belgium | BNL | *Quercus robur* | unknown |
| 82 | CS311 | S/10/41 |  | Belgium | BNL | *Rhododendron sp.* | unknown |
| 83 | CS313 | S/10/47 | coalescent | Belgium | BNL | *Rhododendron sp.* | unknown |
| 84 | CS317 | S/10/74 |  | Belgium | BNL | *Rhododendron sp.* | unknown |
| 85 | CS318 | S/10/83 | coalescent | Belgium | BNL | *Rhododendron sp.* | unknown |
| 86 | CS320 | S/10/86 |  | Belgium | BNL | *Rhododendron sp.* | unknown |
| 87 | CS321 | S/10/87 | coalescent | Belgium | BNL | *Rhododendron sp.* | unknown |
| 88 | CS322 | S/10/99 |  | Belgium | BNL | *Rhododendron sp.* | unknown |
| 89 | CS323 | S/10/104 |  | Belgium | BNL | *Rhododendron sp.* | unknown |
| 90 | CS324 | S/10/105 |  | Belgium | BNL | *Rhododendron sp.* | unknown |
| 91 | CS325 | S/10/111 |  | Belgium | BNL | *Rhododendron sp.* | unknown |
| 92 | CS326 | S/10/112 |  | Belgium | BNL | *Rhododendron sp.* | unknown |
| 93 | CS328 | S/10/122 |  | Belgium | BNL | *Rhododendron sp.* | unknown |
| 94 | CS329 | S/10/150 |  | Belgium | BNL | *Rhododendron sp.* | unknown |
| 95 | CS331 | S/11/121 |  | Belgium | BNL | *Rhododendron sp.* | unknown |
| 96 | CS332 | S/11/122A |  | Belgium | BNL | *Rhododendron sp.* | unknown |
| 97 | CS334 | S/11/154 |  | Belgium | BNL | *Rhododendron sp.* | unknown |
| 98 | CS335 | S/11/155 |  | Belgium | BNL | *Rhododendron sp.* | unknown |
| 99 | CS336 | S/11/156A |  | Belgium | BNL | *Rhododendron sp.* | unknown |
| 100 | CS338 | S/11/212 |  | Belgium | BNL | *Rhododendron sp.* | unknown |
| 101 | CS340 | S/11/225 | coalescent | Belgium | BNL | *Rhododendron sp.* | unknown |
| 102 | CS205 | PD 20025913 - 1 | coalescent | NL | BNL | *Rhododendron sp.* | unknown |
| 103 | CS347 | Serbia 22 |  | Serbia | BAL | *Fraxinus angustifolia & Quercus robur* | soil sample, forest |
| 104 | CS348 | Serbia 28 |  | Serbia | BAL | *Acer pseudoplatanus* | soil sample, forest |
| 105 | CS363 | Serbia 1 |  | Serbia | BAL | *Fraxinus angustifolia* | soil sample, forest |
| 106 | CS364 | Serbia 11 |  | Serbia | BAL | *Quercus petraea* | soil sample, forest |
| 107 | CS366 | Serbia 16 |  | Serbia | BAL | *Fraxinus angustifolia & Quercus robur* | soil sample, forest |
| 108 | CS369 | Serbia 48 |  | Serbia | BAL | *Fagus sylvatica* | soil sample, forest |
| 109 | CS370 | Serbia 3 | coalescent | Serbia | BAL | *Quercus robur & Fraxinus angustifolia* | soil sample, forest |
| 110 | CS537 | CIT 94 |  | Serbia_Kosutnjak | BAL | *Q. petraea* | unknown |
| 111 | CS125 | 79-2 |  | Slovenia_ Haloze | BAL | *Fagus sylvatica* | nat pop |
| 112 | CS131 | 253-3 |  | Slovenia_ Kranj | BAL | *Taxus sp.* | nat pop |
| 113 | CS126 | 497-1 |  | Slovenia_ Ljubljana | BAL | *Fagus sylvatica* | nat pop |
| 114 | CS128 | 590-6 |  | Slovenia_ Ljubljana | BAL | *Fagus sylvatica* | nat pop |
| 115 | CS129 | 590-1 |  | Slovenia_ Ljubljana | BAL | *Fagus sylvatica* | nat pop |
| 116 | CS132 | 607-3 |  | Slovenia_ Ljubljana | BAL | *Fagus sylvatica* | nat pop |
| 117 | CS130 | 315-2 |  | Slovenia_ Mozirje | BAL | *Rhododendron sp.* | nat pop |
| 118 | CS127 | 517-4 |  | Slovenia_ VolcjiPotok | BAL | *Fagus sylvatica* | nat pop |
| 119 | CS224 | P-28 |  | Slovenia_GoriskaBrda | BAL | *Prunus avium* | unknown |
| 120 | CS222 | 340 |  | Slovenia_Kranj | BAL | *Taxus sp. + soil* | unknown |
| 121 | CS220 | 630 |  | Slovenia_Ljubljana | BAL | *Pieris sp.* | unknown |
| 122 | CS221 | 585 | coalescent | Slovenia_Ljubljana | BAL | *Picea omorika.+ soil* | unknown |
| 123 | CS218 | 422-2 |  | Slovenia_VolcjiPotok | BAL | *Rhododendron sp.* | unknown |
| 124 | CS219 | 422-7 |  | Slovenia_VolcjiPotok | BAL | *Magnolia sp.* | unknown |
| 125 | CS223 | P-M2 |  | Slovenia_VolcjiPotok | BAL | *Magnolia sp. + soil* | unknown |
| 126 | CS471 | P 139.07 |  | CZ_Brezinka_Bohemia | EEU | *Alnus glutinosa* | bark/collar rot |
| 127 | CS482 | P 474.11 | coalescent | CZ_CeskeBudejovice_Bohemia | EEU | *Acer pseudoplatanus* | bark/collar rot |
| 128 | CS467 | P 035.06 |  | CZ_Hvezdonice_Bohemia | EEU | *Rhododendron sp.* | bark/collar rot |
| 129 | CS472 | P 162.07 |  | CZ_Jevany_Bohemia | EEU | *Rhododendron sp.* | leaf/anthracnose |
| 130 | CS479 | P 354.09 |  | CZ_Lysa_Bohemia | EEU | *Tilia cordata* | rhizosphere/root rot |
| 131 | CS474 | P 215.08 |  | CZ_Ostra_Bohemia | EEU | *Tilia cordata* | rhizosphere/root rot |
| 132 | CS473 | P 185.07 |  | CZ_Praha | EEU | *Fraxinus excelsior* | rhizosphere/root rot |
| 133 | CS475 | P 232.08 |  | CZ_Praha | EEU | *Acer pseudoplatanus* | rhizosphere/root rot |
| 134 | CS470 | P 127.07 |  | CZ_Pruhonice_ Bohemia | EEU | *Vaccinium sp.* | leaf/anthracnose |
| 135 | CS481 | P 410.10 |  | CZ_TisovaHlinne_Bohemia | EEU | *Fraxinus excelsior* | rhizosphere/root rot |
| 136 | CS469 | P 070.07 |  | CZ_Trebon_Bohemia | EEU | *Quercus robur* | rhizosphere/root rot |
| 137 | CS478 | P 306.09 |  | CZ_Troubky_Moravia | EEU | *Fraxinus excelsior* | rhizosphere/root rot |
| 138 | CS066 | P86/04 |  | Hungary | EEU | *Alnus glutinosa* soil | forest |
| 139 | CS375 | JA222 |  | Hungary_Velem | EEU | *Alnus-Carpinus* forest soil/alder bait | forest |
| 140 | CS377 | JA211 |  | Hungary_Csehi | EEU | *Alnus* forest soil/*Syringa vulgaris* bait | forest |
| 141 | CS388 | JA309 |  | Hungary_Szombathely | EEU | *Rhododenron sp*., shoot bark | ornamental garden |
| 142 | CS390 | P170 |  | Hungary_Várpalota | EEU | *Fraxinus sp*., soil/cherry laurel leaf bait | forest |
| 143 | CS391 | JA456 |  | Hungary_Cák | EEU | *Salix-Ald*er forest soil/*Rhododendron* bait | forest |
| 144 | CS394 | P171 |  | Hungary_Várpalota | EEU | *Fraxinus sp*., soil/cherry laurel leaf bait | forest |
| 145 | CS396 | JA420 |  | Hungary_Velem | EEU | *Fagus-Quercus* forest soil/*Rhododendron* bait | forest |
| 146 | CS399 | JA394 |  | Hungary_Gencsapáti | EEU | *Abies procera*, stem collar bark | ornamental nursery |
| 147 | CS406 | H-92/04 |  | Hungary_Úrkút | EEU | *Fagus sylvatica*, stem bark | forest |
| 148 | CS407 | JA546 | coalescent | Hungary_Ják | EEU | young forest soil/*Rhododendron* bait | forest |
| 149 | CS410 | JA454 |  | Hungary_Cák | EEU | *Salix-Alder* forest soil/cherry laurel bait | forest |
| 150 | CS412 | P318 |  | Hungary_unknown | EEU | *Alnus glutinosa* soil/cherry laurel leaf bait | forest |
| 151 | CS413 | P85/04 |  | Hungary_Csehi | EEU | *Alnus glutinosa* root with little soil/cherry-laurel leaf bait | forest |
| 152 | CS275 | 165/1 |  | Hungary_Kapuvar | EEU | black walnut | soil |
| 153 | CS276 | 166/1 |  | Hungary_Kapuvar | EEU | black walnut | soil |
| 154 | CS277 | 177 |  | Hungary_Kapuvar | EEU | black walnut | soil |
| 155 | CS371 | H1/02 |  | Hungary_Ocsa | EEU | *A. glutinosa,* forest soil/chery laurel bait | forest |
| 156 | CS278 | 139/1 |  | Hungary_Sarvar | EEU | black walnut | soil |
| 157 | CS279 | 141/1 | coalescent | Hungary_Sarvar | EEU | black walnut | soil |
| 158 | CS280 | 144/1 |  | Hungary_Sarvar | EEU | black walnut | soil |
| 159 | CS018 | BBA 64974 |  | Poland | EEU | *Rhododendron spp.* | unknown |
| 160 | CS061 | BBA 64975 |  | Poland | EEU | *Rhododendron spp.* | unknown |
| 161 | CS196 | 26 |  | Poland | EEU | *Rhododendron* | nursery |
| 162 | CS197 | 136 |  | Poland | EEU | *Fagus sylvatica* | nursery |
| 163 | CS003 | BBA 1494 |  | Germany | D | *Rhododendron simsii* | nursery |
| 164 | CS004 | BBA 15/01-17b |  | Germany | D | *Rhododendron ’Roseum Elegans’* | nursery |
| 165 | CS008 | BBA 64705 |  | Germany | D | *Rhododendron simsii ‘Friedhelm Scherrer‘* | nursery |
| 166 | CS012 | BBA 17/02 |  | Germany | D | *Rhododendron spp.* | nursery |
| 167 | CS016 | BBA (8)1 |  | Germany | D | *A. hippocastanum*, soil & root mix | private garden |
| 168 | CS019 | BBA 15/01-31b | coalescent | Germany | D | *Rhododendron minus* | nursery |
| 169 | CS020 | BBA 1015/1 |  | Germany | D | *Rhododendron simsii* | nursery |
| 170 | CS021 | BBA 1132/1/1 |  | Germany | D | *Rhododendron simsii ’Knut Erwen’* | nursery |
| 171 | CS025 | BBA 1763 |  | Germany | D | *Rhododendron simsii ‘Nanu‘* | nursery |
| 172 | CS027 | BBA 18/97-5 |  | Germany | D | *Genista spp.* | nursery |
| 173 | CS028 | BBA 1126/1/1 |  | Germany | D | *Rhododendron simsii* | nursery |
| 174 | CS037 | BBA 15/01-45a1 |  | Germany | D | *Rhododendron spp.* | nursery |
| 175 | CS041 | BBA (8)2A | coalescent | Germany | D | *A. hippocastanum*, soil & root mix | private garden |
| 176 | CS042 | BBA 31/04-1.1a |  | Germany | D | *F. sylvatica* ‘Atropunicea’ | private garden |
| 177 | CS043 | BBA 2/95-1 |  | Germany | D | *Rhododendron spp.* | nursery |
| 178 | CS044 | BBA 15/01-32a |  | Germany | D | *Rhododendron minus* | nursery |
| 179 | CS045 | BBA 31/04-3.1b |  | Germany | D | *F. sylvatica* ‘Atropunicea’ | private garden |
| 180 | CS047 | BBA 54/05-2.1 |  | Germany | D | *Rhododendron sp.,* soil | historic garden |
| 181 | CS048 | BBA 1538a |  | Germany | D | *Erica gracilis* | nursery |
| 182 | CS053 | BBA 15/01-17c |  | Germany | D | *Rhododendron ’Roseum Elegans’* | nursery |
| 183 | CS059 | BBA 44/05-14 |  | Germany | D | *Rhododendron ‘Maharani’* | nursery |
| 184 | CS062 | BBA 4/97-1 |  | Germany | D | *A. hippocastanum*, soil & root mix | historic garden |
| 185 | CS068 | BBA (8)2B | coalescent | Germany | D | *A. hippocastanum*, soil & root mix | private garden |
| 186 | CS069 | BBA 1514b |  | Germany | D | *Calluna spp.* | nursery |
| 187 | CS070 | BBA 44/05-15 |  | Germany | D | *Rhododendron spp. hybr.* | nursery |
| 188 | CS071 | BBA 15/01-45b3 |  | Germany | D | *Rhododendron spp.* | nursery |
| 189 | CS072 | BBA 44/05-13 |  | Germany | D | *Rhododendron yakushimanum ‘Percy Wiseman’* | nursery |
| 190 | CS078 | BBA 13/95-3.2b |  | Germany | D | *Alnus glutinosa* | forest |
| 191 | CS079 | BBA 37/04-3B-3 |  | Germany | D | *Alnus incana,* soil | river bank |
| 192 | CS080 | BBA 1015/2 |  | Germany | D | *Rhododendron simsii* | nursery |
| 193 | CS084 | BBA 15/01-27d |  | Germany | D | *Rhododendron ’Diadem’* | nursery |
| 194 | CS133 | BBA 1538b |  | Germany | D | *Calluna vulgaris ‘Carmen‘* | nursery |
| 195 | CS139 | BBA 15/01-27f |  | Germany | D | *Rhododendron ’Diadem’* | nursery |
| 196 | CS546 | CIT 173 |  | Germany_Albaching | D | *F. sylvatica* | unknown |
| 197 | CS552 | CIT 200 |  | Germany_Brannenburg | D | *F. sylvatica* | soil |
| 198 | CS529 | CIT 36 |  | Germany_Bavaria | D | *F. sylvatica* | Bot Garden, soil |
| 199 | CS560 | CIT 223 |  | Germany_Flintsbach | D | *F. sylvatica* | collar rot |
| 200 | CS456 | Quercus 1 b |  | Germany_Freising | D | *Quercus sp.* | Radici quercia |
| 201 | CS531 | CIT 55 |  | Germany_Garmisch | D | *F. sylvatica* | soil |
| 202 | CS556 | CIT 216 |  | Germany_Grainbach | D | *F. sylvatica* | collar rot |
| 203 | CS541 | CIT 125 |  | Germany_Herrenchiemsee | D | *F. sylvatica* | canker |
| 204 | CS555 | CIT 210 | coalescent | Germany_Heuberg | D | *F. sylvatica* | unknown |
| 205 | CS550 | CIT 193 |  | Germany_Hoegelwald | D | *F. sylvatica* | canker |
| 206 | CS547 | CIT 178 |  | Germany_Irschenberg | D | *F. sylvatica* | unknown |
| 207 | CS558 | CIT 220 |  | Germany_Irschenberg | D | *F. sylvatica* | root necrosis |
| 208 | CS559 | CIT 221 |  | Germany_Irschenberg | D | *F. sylvatica* | canker |
| 209 | CS274 | Bu16 |  | Germany_Konstanz | D | *Fagus sp.* seedling | forest |
| 210 | CS545 | CIT 153 |  | Germany_Kreutsee | D | *F. sylvatica* | canker |
| 211 | CS557 | CIT 219 |  | Germany_NeustadtAisch | D | *F. sylvatica* | soil |
| 212 | CS554 | CIT 209 |  | Germany_Nussdorf | D | *F. sylvatica* | canker |
| 213 | CS553 | CIT 202 |  | Germany_Samerberg | D | *F. sylvatica* | collar rot |
| 214 | CS543 | CIT 127 |  | Germany_Schernfeld | D | *F. sylvatica* | collar rot |
| 215 | CS544 | CIT 140 |  | Germany_StMargarethen | D | *F. sylvatica* | collar rot |
| 216 | CS535 | CIT 92 | coalescent | Turkey | TK | *Quercus sp.* | soil |
| 217 | CS120 | 2 M-8 |  | Turkey_ Caycuma | TK | oak soil forest | forest |
| 218 | CS121 | 6 M-4 |  | Turkey_ Caycuma | TK | oak soil forest | forest |
| 219 | CS122 | 29 Sa-Ks-1 |  | Turkey_ Samsun | TK | chestnut forest soils | forest |
| 220 | CS123 | 31 Si-Ks-4 |  | Turkey_ Sinop | TK | chestnut forest soils | forest |
| 221 | CS124 | 40 Beldibi M-19 |  | Turkey_ Zonguldak | TK | unknown | unknown |

**Table B:** Origin, gene bank accession numbers and supporting information on the 37 *Phytophthora plurivora* isolates used in the coalescent analyses.

| **Strain** | **_ITS** | **Enolase** | **Btub** | **Cox I** | **HSP90** | **Tig A** | **Trp 1** | **Alternate ID** | **Isolated** | **Country** | **Location** | **Isolated By** | **Host genus species** |
| --- | --- | --- | --- | --- | --- | --- | --- | --- | --- | --- | --- | --- | --- |
| CS019 | KF443843 | KF443884 | KF443900 | KF443919 | none** | KF443992 | KF444038 | BBA 15/01-31b | 2001 | Germany | unknown | Sabine Werres | *Rhododendron minus* |
| CS038 | KF443848 | KF443878 | KF443893 | KF443943 | KF443950 | KF443997 | KF444039 | BBA 1514a | 1986 | Germany | unknown | Sabine Werres | *Erica spp. hybr.* |
| CS041 | KF443847 | KF443883 | KF443904 | KF443926 | KF443949 | KF443993 | none | BBA (8)2A | 1987 | Germany | unknown | Sabine Werres | soil & root mix (*A. hippocastanum*) |
| CS068 | KF443834 | KF443862 | KF443901 | KF443941 | KF443971 | KF443985 | KF444029 | BBA (8)2B | 1987 | Germany | unknown | Sabine Werres | soil & root mix (*A. hippocastanum* ) |
| CS073 | KF443844 | KF443859 | KF443887 | KF443918 | KF443967 | KF444010 | KF444021 | 273 | unknown | Switzerland | Geneve | Agroscope, CH | *Acer platanoides* |
| CS077 | KF443845 | KF443854 | KF443902 | KF443928 | KF443973 | KF443990 | KF444017 | 113 | unknown | Switzerland | Angers | Agroscope, CH | unknown |
| CS096 | KF443846 | KF443880 | KF443903 | none* | none | KF443998 | none | PI33/ M5614 | 2006 | Switzerland | unknown | D. Rigling, WSL | *Rhododendron 'Roseum elegans'* |
| CS114 | KF443824 | KF443881 | KF443898 | KF443940 | KF443963 | KF443991 | KF444041 | 174 | unknown | Switzerland | Brisago Island, Ticcino | Agroscope, CH | *Rhododendron sp.,* soil |
| CS143 | KF443841 | KF443860 | KF443905 | KF443927 | KF443952 | KF444000 | KF444034 | 450 | 2008 | Finland | unknown | Arja Lilja | *Syringa* |
| CS144 | KF443836 | KF443849 | KF443912 | KF443930 | KF443961 | KF444014 | KF444019 | 444 | 2007 | Finland | unknown | Arja Lilja | unknown |
| CS156 | KF443839 | KF443850 | KF443897 | KF443923 | KF443955 | KF444001 | KF444040 | PC09-2097 | 2009 | USA | unknown | Wendy Sutton | unknown |
| CS173 | KF443837 | KF443856 | KF443894 | KF443929 | KF443980 | KF444012 | none | 120-W-1.17 | 2010 | USA | unknown | Wendy Sutton | Stream |
| CS180 | KF443838 | KF443879 | KF443895 | KF443925 | KF443951 | KF443995 | KF444037 | 05-02 | 2002 | USA | unknown | Wendy Sutton | Nursery Foliage |
| CS205 | KF443842 | KF443863 | KF443906 | KF443931 | KF443979 | KF444009 | none | PD 20025913-1 | unknown | Netherlands | unknown | Johan Meffert | *Rhododendron sp.* |
| CS221 | KF443840 | KF443858 | KF443885 | KF443944 | KF443957 | none | none | 585 | 2009 | Slovenia | Ljubiliana | M. Žerjav | *Picea omorika*, soil |
| CS256 | KF443813 | KF443857 | KF443913 | KF443924 | KF443972 | KF444006 | KF444026 | JP-07-0310 | 2007 | USA | Oregon | Parke, Grunwald | *Rhododendron sp.* |
| CS258 | KF443833 | KF443852 | KF443896 | KF443920 | KF443965 | KF443996 | KF444022 | PCT-07-015 | 2007 | USA | Oregon | Osterbauer, Grunwald | *Rhododendron pink* |
| CS260 | KF443814 | KF443882 | KF443892 | none | KF443948 | KF444005 | KF444036 | JP-08-113 | 2008 | USA | Oregon | Parke, Grunwald | *Pieris japonica* |
| CS264 | KF443816 | none | KF443911 | none | KF443975 | KF444008 | KF444025 | PCT-09-003 | 2009 | USA | Oregon | Osterbauer, Grunwald | unknown |
| CS279 | KF443818 | KF443853 | KF443886 | KF443946 | KF443960 | none | KF444028 | 141/1 | 2011 | Hungary | Sárvár | Judit Kovacs | black walnut |
| CS303 | KF443820 | KF443864 | KF443914 | KF443922 | KF443982 | KF444002 | KF444015 | CIT3 | 2000 | France | Bazoilles/Meuse | Claude Husson | *Alnus glutinosa* |
| CS306 | KF443830 | KF443861 | KF443916 | KF443921 | KF443978 | KF444003 | none | PHY8.5 | 2008 | France | Baerendorf | Claude Husson | *Alnus glutinosa* |
| CS308 | KF443835 | KF443855 | KF443888 | KF443939 | KF443977 | KF443988 | none | CIT1 | 2002 | France | Plouigneau | Ioos & Husson | *Rhododendron cv Scintillation* |
| CS313 | KF443815 | KF443851 | KF443889 | KF443947 | KF443970 | KF444007 | KF444024 | S/10/47 | 2010 | Belgium | unknown | Kurt Heugens | *Rhododendron sp.* |
| CS318 | KF443821 | KF443868 | KF443910 | KF443936 | KF443964 | KF443989 | KF444027 | S/10/83 | 2010 | Belgium | unknown | Kurt Heugens | *Rhododendron sp.* |
| CS321 | KF443823 | KF443871 | KF443890 | KF443933 | KF443958 | none** | KF444035 | S/10/87 | 2010 | Belgium | unknown | Kurt Heugens | *Rhododendron sp.* |
| CS340 | KF443817 | KF443867 | KF443891 | KF443938 | KF443962 | KF444011 | KF444031 | S/11/225 | 2011 | Belgium | unknown | Kurt Heugens | *Rhododendron sp.* |
| CS370 | KF443822 | KF443873 | KF443909 | KF443935 | KF443968 | none | KF444032 | Serbia 3 | 2011 | Serbia | soil sample | Ivan Milencovic | *Q. robur & F. angustifolia* |
| CS393 | KF443812 | KF443872 | KF443915 | KF443942 | KF443974 | KF443984 | none | CBS124087 | 1994 | Germany | Bavaria | CBS | unknown |
| CS407 | KF443831 | KF443869 | none | none | KF443976 | none | none | JA546 | Jun 05 | Hungary | unknown | Jozsef Bakonyi | unknown |
| CS427 | KF443825 | KF443876 | none | KF443932 | KF443959 | KF443986 | KF444020 | 6G3T9.5(7°) | 2008 | Italy | Carb. | Andrea Vanini | *Castanea sativa* |
| CS482 | KF443827 | KF443866 | none | KF443945 | KF443953 | none | KF876011 | P 474.11 | 2011 | Czech Republic | Bohemia | Matej Panek | *Acer pseudoplatanus* |
| CS535 | KF443832 | KF443877 | none | KF443917 | KF443981 | KF444004 | KF444023 | CIT 92 | unknown | Turkey | unknown | F. Fleischmann | *Quercus sp.,* soil |
| CS555 | KF443819 | KF443870 | none | KF443937 | KF443969 | KF443983 | KF444030 | CIT 210 | unknown | Germany | Bavaria | F. Fleischmann | *F. sylvatica* |
| CS608 | KF443828 | KF443874 | KF443907 | none | KF443966 | KF443987 | KF444016 | W.004 | unknown | USA | Wisconsin | Steve Jeffers | *Acer saccharum* |
| CS611 | KF443829 | KF443865 | KF443908 | none* | KF443956 | KF444013 | KF444018 | W.019 | unknown | USA | Wisconsin | Steve Jeffers | *Rhododendron x catawbiense* |
| CS622 | KF443826 | KF443875 | KF443899 | KF443934 | KF443954 | KF443994 | KF444033 | 189 | unknown | Switzerland | Vaud | Agroscope, CH | apple |

none - no sequence available, due to bad amplification; not used for analysis, * contains stop codon - not uploaded to GenBank, ** contains indel - not uploaded to GenBank

**Table C:** F_ST_ values between all population pairs of European and US *Phytophthora plurivora isolates*. Populations are abbreviated as follows: Italy (I), Finland (FIN), United Kingdom (UK), US East Coast (EC), Austria and Switzerland (Alps), US West Coast (WC), France (F), Belgium and the Netherlands (BNL), Serbia and Slovenia (Balkans, BAL), Czech Republic, Hungary and Poland (Eastern Europe, EEU), Germany (D) and Turkey (TR). Significance intervals: none of the values were statistically significant (p < 0.05).

|  | I | FIN | UK | EC | Alps | WC | F | BNL | BAL | EEU | D |
| --- | --- | --- | --- | --- | --- | --- | --- | --- | --- | --- | --- |
| FIN | 0.22 |  |  |  |  |  |  |  |  |  |  |
| UK | 0.03 | 0.11 |  |  |  |  |  |  |  |  |  |
| EC | 0.17 | 0.30 | 0.10 |  |  |  |  |  |  |  |  |
| Alps | 0.10 | 0.09 | 0.06 | 0.06 |  |  |  |  |  |  |  |
| WC | 0.18 | 0.26 | 0.20 | 0.07 | 0.13 |  |  |  |  |  |  |
| F | 0.08 | 0.22 | 0.14 | 0.14 | 0.14 | 0.10 |  |  |  |  |  |
| BNL | 0.10 | 0.16 | 0.01 | 0.08 | 0.11 | 0.18 | 0.17 |  |  |  |  |
| BAL | 0.06 | 0.15 | 0.06 | 0.05 | 0.00 | 0.11 | 0.12 | 0.10 |  |  |  |
| EEU | 0.06 | 0.11 | 0.06 | 0.06 | 0.05 | 0.09 | 0.03 | 0.11 | 0.05 |  |  |
| D | 0.11 | 0.14 | 0.05 | 0.08 | 0.02 | 0.17 | 0.15 | 0.09 | 0.02 | 0.06 |  |
| TR | 0.22 | 0.37 | 0.36 | 0.37 | 0.22 | 0.24 | 0.16 | 0.38 | 0.14 | 0.19 | 0.24 |

**Table D:** Supplementary information on the *Phytophthora plurivora* isolates used in the Haplotype Network analysis.

| **Strain ID** | **Country** | **Btub_ HT** | **CoxI_ HT** | **Enolase_ HT** | **HSP90_ HT** | **ITS_HT** | **TigA_HT** | **Trp1_HT** |
| --- | --- | --- | --- | --- | --- | --- | --- | --- |
| CS019 | D | btub_HT1 | coxI_HT1 | enoalse_HT1 | HSP90_HT1 | ITS_HT1 | TigA_HT1 | trp1_HT1 |
| CS038 | D | btub_HT1 | coxI_HT1 | enoalse_HT1 | HSP90_HT1 | ITS_HT2 | TigA_HT1 | trp1_HT1 |
| CS041 | D | btub_HT1 | coxI_HT5 | enoalse_HT1 | HSP90_HT1 | ITS_HT1 | TigA_HT1 | n.a. |
| CS068 | D | btub_HT1 | coxI_HT1 | enoalse_HT1 | HSP90_HT1 | ITS_HT1 | TigA_HT1 | trp1_HT1 |
| CS073 | CH | btub_HT1 | coxI_HT3 | enoalse_HT1 | HSP90_HT1 | ITS_HT1 | TigA_HT5 | trp1_HT1 |
| CS077 | D | btub_HT1 | coxI_HT1 | enoalse_HT1 | HSP90_HT1 | ITS_HT1 | TigA_HT1 | trp1_HT1 |
| CS096 | CH | btub_HT1 | n.a. | enoalse_HT1 | n.a. | ITS_HT1 | TigA_HT1 | trp1_HT1 |
| CS114 | CH | btub_HT1 | coxI_HT1 | enoalse_HT2 | HSP90_HT1 | ITS_HT1 | TigA_HT1 | trp1_HT1 |
| CS143 | FIN | btub_HT1 | coxI_HT1 | enoalse_HT1 | HSP90_HT1 | ITS_HT1 | TigA_HT2 | trp1_HT1 |
| CS144 | FIN | btub_HT1 | coxI_HT1 | enoalse_HT1 | HSP90_HT1 | ITS_HT1 | TigA_HT5 | trp1_HT1 |
| CS156 | USA_OR | btub_HT1 | coxI_HT2 | enoalse_HT1 | HSP90_HT1 | ITS_HT1 | TigA_HT2 | trp1_HT1 |
| CS173 | USA_OR | btub_HT1 | coxI_HT1 | enoalse_HT1 | HSP90_HT3 | ITS_HT3 | TigA_HT6 | trp1_HT1 |
| CS180 | USA_OR | btub_HT1 | coxI_HT2 | enoalse_HT1 | HSP90_HT1 | ITS_HT1 | TigA_HT1 | trp1_HT1 |
| CS205 | NL | btub_HT1 | coxI_HT1 | enoalse_HT1 | HSP90_HT3 | ITS_HT1 | TigA_HT7 | n.a. |
| CS221 | SLO | btub_HT3 | coxI_HT1 | enoalse_HT1 | HSP90_HT1 | ITS_HT1 | n.a. | n.a. |
| CS256 | USA_OR | btub_HT1 | coxI_HT2 | enoalse_HT1 | HSP90_HT1 | ITS_HT1 | TigA_HT7 | trp1_HT1 |
| CS258 | USA_OR | btub_HT1 | coxI_HT2 | enoalse_HT1 | HSP90_HT1 | ITS_HT1 | TigA_HT1 | trp1_HT1 |
| CS260 | USA_OR | btub_HT1 | n.a. | enoalse_HT1 | HSP90_HT1 | ITS_HT1 | TigA_HT5 | trp1_HT1 |
| CS264 | USA_OR | btub_HT1 | n.a. | enoalse_HT1 | HSP90_HT1 | ITS_HT1 | TigA_HT7 | trp1_HT1 |
| CS279 | H | btub_HT1 | coxI_HT1 | enoalse_HT1 | HSP90_HT1 | ITS_HT1 | TigA_HT2 | trp1_HT1 |
| CS303 | F | btub_HT2 | coxI_HT4 | enoalse_HT1 | HSP90_HT5 | ITS_HT4 | TigA_HT3 | trp1_HT3 |
| CS306 | F | btub_HT2 | coxI_HT4 | enoalse_HT1 | HSP90_HT4 | ITS_HT5 | TigA_HT4 | n.a. |
| CS308 | F | btub_HT1 | coxI_HT1 | enoalse_HT1 | HSP90_HT2 | ITS_HT1 | TigA_HT1 | n.a. |
| CS313 | B | btub_HT1 | coxI_HT1 | enoalse_HT1 | HSP90_HT1 | ITS_HT1 | TigA_HT7 | trp1_HT1 |
| CS318 | B | btub_HT1 | coxI_HT1 | enoalse_HT1 | HSP90_HT1 | ITS_HT1 | TigA_HT1 | trp1_HT1 |
| CS321 | B | btub_HT4 | coxI_HT1 | enoalse_HT1 | HSP90_HT1 | ITS_HT1 | TigA_HT1 | trp1_HT1 |
| CS340 | B | btub_HT1 | coxI_HT1 | enoalse_HT1 | HSP90_HT1 | ITS_HT1 | TigA_HT5 | trp1_HT1 |
| CS370 | SER | btub_HT1 | coxI_HT1 | enoalse_HT1 | HSP90_HT1 | ITS_HT1 | n.a. | trp1_HT1 |
| CS393 | D | btub_HT2 | coxI_HT4 | enoalse_HT1 | HSP90_HT1 | ITS_HT1 | TigA_HT1 | n.a. |
| CS407 | H | n.a. | coxI_HT1 | enoalse_HT1 | HSP90_HT6 | ITS_HT5 | n.a. | n.a. |
| CS427 | I | n.a. | coxI_HT1 | n.a. | HSP90_HT1 | ITS_HT1 | TigA_HT1 | trp1_HT1 |
| CS482 | CZ | n.a. | coxI_HT6 | enoalse_HT1 | HSP90_HT1 | ITS_HT1 | n.a. | trp1_HT2 |
| CS535 | TK | n.a. | coxI_HT4 | enoalse_HT1 | HSP90_HT5 | ITS_HT5 | TigA_HT4 | trp1_HT1 |
| CS555 | D | n.a. | coxI_HT1 | enoalse_HT1 | HSP90_HT1 | ITS_HT1 | TigA_HT1 | trp1_HT1 |
| CS608 | USA_EC | btub_HT1 | n.a. | enoalse_HT1 | HSP90_HT1 | ITS_HT1 | TigA_HT1 | trp1_HT1 |
| CS611 | USA_EC | btub_HT1 | coxI_HT6 | enoalse_HT1 | HSP90_HT1 | ITS_HT1 | TigA_HT5 | trp1_HT1 |
| CS622 | CH | btub_HT1 | coxI_HT1 | enoalse_HT1 | HSP90_HT1 | ITS_HT3 | TigA_HT1 | trp1_HT1 |

HT – haplotype; n.a. – not analyzed;

Germany (D), Switzerland (CH), Finland (FIN), USA Oregon (USA_OR), Netherlands (NL), Slovenia (SLO), Hungary (H), France (F), Belgium (B), Serbia (SRB), Italy (I), Czech Republic (CZ), Turkey (TR), US East Coast (USA_EC);
