# Supplementary figures and images for: Population History and Pathways of Spread of the Plant Pathogen Phytophthora plurivora
Source: PLoS One. 2014 Jan 10;9(1):e85368. doi: 10.1371/journal.pone.0085368 (PMC3888410; doi:10.1371/journal.pone.0085368)

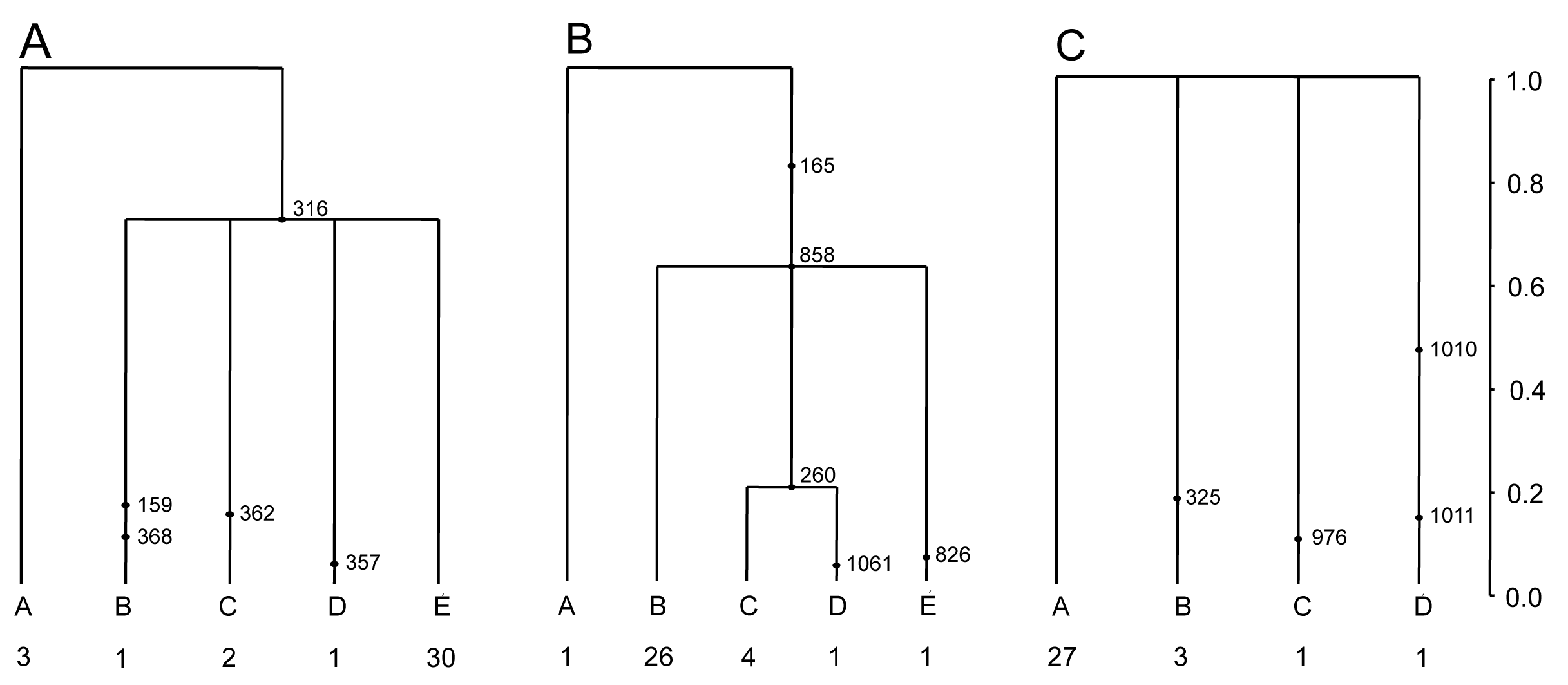

Supplement: Figure S1 — Gene genealogy for Phytophthora plurivora. Coalescent-based gene genealogy for the three genes (a) ITS (37 sequences), (b) cox I (33 sequences) and (c) btub (32 sequences) generated using GENETREE [67], which assumes no recombination. Each genealogy is scaled to time to the most recent common ancestor (TMRCA) of 1.0 for each locus. Timescale is in coalescent units of effective population size. Mutations are labelled by their location in the sequence. Letters represent haplotypes and numbers underneath indicate the number of isolates that share that specific haplotype. The ancestral haplotype was found in (a) France, Hungary and Turkey and (b) Germany, while for bub (c) all haplotypes diverged at the same time. For (c) the oldest haplotype was selected by GENETREE based on likelihood scores. Note: At position 824 of the cox I region there was a incompatible site, leading to 5 haplotypes above, while for the analyses in Fig. 2 and Table 3 we indicate 6 haplotypes. (TIF) [file pone.0085368.s002.tif]
